# Stroke in Traditional Korean Medicine: A Nine-Year Multicentre Community-Based Study in South Korea

**DOI:** 10.1038/srep28286

**Published:** 2016-06-22

**Authors:** Mi Mi Ko, Ju Ah Lee, Min Ho Cha, Byoung-Kab Kang, Myeong Soo Lee

**Affiliations:** 1 KM Fundamental Research Division, Korea Institute of Oriental Medicine, Daejeon 305-811, South Korea; 2 Clinical Research Division, Korea Institute of Oriental Medicine, Daejeon 305-811, South Korea

## Abstract

In Korea, patients with stroke are commonly treated using traditional Korean medicine (TKM). The aim of this study was to provide information on the clinical characteristics of the pattern identification (PI) of stroke used in TKM. Stroke patients admitted to 15 TKM university hospitals from April 2005 through December 2013 were evaluated. The measured variables included the following factors as they related to the PI: (a) stroke etiology; (b) distribution of symptoms/signs; (c) physical characteristics and lifestyle parameters; (d) medical history; and (e) stroke-related laboratory results. Among 4912 stroke patients, 3466 patients received the same PI by two experts with the following distribution: Qi-Deficiency pattern (n = 810), Fire-Heat (FH) pattern (n = 1031), Dampness-Phlegm (DP) pattern (n = 1127), and Yin-Deficiency pattern (n = 498). Approximately 89.9% of subjects enrolled in this study had cerebral infarction. Some of specific symptoms were related to each type of PI, and obese phenotypes and blood lipids were significantly related to DP and FH. These results showed the characteristics of each type of PI and should lead to the standardization of diagnosis for stroke in TKM.

In Korea, the second most common cause of death is stroke, and the treatment and management of cerebrovascular diseases incurs a large financial burden^1^,^2^. Many stroke patients receive traditional medical care in Korea. According to a recent report from the Korean Ministry of Health and Welfare, stroke inpatients comprise the largest proportion of inpatients in traditional Korean medicine (TKM) hospitals^3^.

PI is a traditional diagnostic system characterized by its own theoretical basis and practical experience in traditional Eastern-Asian medicine (TEAM), which is practiced in China, Korea and Japan^4^,^5^,^6^,^7^. PI is the foundation of the treatment disease in TEAM. In traditional Chinese medicine (TCM), stroke is classified into six subtypes according to PI, and Zhou *et al*. reported the efficacy of therapies based on PI among patients with cerebral infarction^8^.

PI diagnosis is processed by the combination of signs and symptoms that are, specific and/or non-specific for diseases through observation, listening, questioning and feeling the pulse^9^.

However, the PI diagnosis exhibits limited objectivity and reproducibility due to the lack of standardized measurement indices, and it is difficult to objectify a diagnosis among physicians due to variations in their knowledge and experience^4^,^5^,^6^,^10^. As the demand for the reestablishment and development of TKM has increased, studies on the establishment of a scientific basis for and the standardization of PI have been actively conducted^7^.

The Korea Institute of Oriental Medicine (KIOM) conducted the project “Fundamental study for the standardization and objectification of pattern identification in TKM for stroke (SOPI-Stroke)” over the course of nine years from April 2005 to December 2013^5^,^6^.

The purpose of this paper was to evaluate the distribution and characteristics of several key factors, including demographic parameters, lifestyle factors, symptoms/signs, stroke etiology, medical history, and laboratory results, in relation to the PI of stroke patients in TKM according to the results of the SOPI-Stroke project.

## Results

### Stroke etiology in relation to PI

Of the 4921 stroke patients collected over nine years, 3466 patients received the same PI diagnosis by two TKM experts with the following distribution: Qi-Deficiency (QD) pattern (n = 810, 23.37%), Fire-Heat (FH) pattern (n = 1031, 29.75%), Dampness-Phlegm (DP) pattern (n = 1127, 32.52%), and Yin-Deficiency (YD) pattern (n = 498, 14.37%). The different stroke types and NIHSS scores in relation to PI are presented in Table 1. Approximately 89.9% of subjects enrolled in this study had cerebral infarction (CI). In the TOAST classification of CI type, many subjects exhibited small vessel occlusion (SVO) rather than large artery atherosclerosis (LAA). The NIHSS score of the majority of patients was below 15. Among PI, the frequency of CI and SVO in the DP pattern was slightly higher than the YD pattern, but the NIHSS score among PI was not different.

### Distribution of symptoms/signs in relation to PI

The Korean standard PI (K-SPI) of stroke consists of four PIs (QD, FH, DP, and YP pattern), and a total of 44 symptoms/signs for determining PI were reported in a previous study^6^. Variables are classified into four types: 19 FH variables, 7 DP variables, 11 QD variables, and 11 YD variables. Table 2 shows the distribution of the variables according to PI. In the FH pattern, headache-like flush, red tongue and strong pulse were the major symptoms. In the DP pattern, obesity (bi-sup) and a white “fur” on the tongue were common. Appearing powerless and lethargic and weak pulse symptoms were commonly observed in the QD pattern. Tidal fever, dry mouth and gauntness were more common in the YD pattern than in other PIs.

### Physical characteristics and lifestyle parameters in relation to PI

Table 3 shows the physical characteristics and lifestyle patterns in relation to PIs. The BMI and WHR, which are related with obesity, were higher in the DP pattern than in other PIs. For lifestyle patterns, subjects that were currently smokers and drinkers were more likely to exhibit the FH pattern than the QD or YD pattern. Food preferences were also different according to PI.

### Medical history in relation to PI

The subjects’ medical histories are presented in Table 4. With regard to past medical history, more than 58% of the patterns showed hypertension, which was the most common (58.43–63.35%) disease. A stroke-related history of diseases such as hypertension, DM, and heart attack was observed. However, the distribution among PIs was not different.

### Blood parameters in relation to PI

The subjects’ blood parameters are presented in Table 5. In the haematology category, the levels of WBC, hemoglobin and hematocrit were higher in the FH pattern than in the other PIs. The platelet level was higher in the DP pattern, and the serum levels of lipids, including total cholesterol and triglycerides, were higher in the DP pattern compared to the other groups. Homocysteine and vitamin B12, which are indirect indicators of stroke, showed a different tendency among the pattern groups. The levels of homocysteine were higher in the FH group compared to the other groups, but the level of vitamin B12 was higher in the YD group.

## Discussion

In the practice of TEAM, including TCM and TKM, a unique decision-making process called Bian Zheng Lun Zhi (PI or syndrome differentiation followed by treatment) is widely used^11^. This method, also called traditional Chinese medical diagnostics, is the procedure and practice of examining patients, determining diseases and differentiating syndromes/identifying patterns of signs and symptoms of diseases^4^. Through the comprehensive analysis of symptoms and signs, which has implications in determining the cause, nature and location of the illness and the patient’s physical condition, their treatment is determined and confirmed^4^,^11^. According to TEAM theory, even patients with the same disease receive different treatments based on PI results. Although this diagnostic system has many advantages in that it uses a comprehensive analysis of symptoms and signs to assess the cause of the diseases, there are many variations in the diagnostic process^7^,^12^.

KIOM focuses on the importance of standardization in PI and has been engaged in relevant research^7^. The SOPI-Stroke project was conducted by KIOM from April 2005 to December 2013 to standardize and objectify PI for stroke through a scientific process^5^,^6^,^10^. In a previous study, our team described the four standard patterns, FH pattern, DP pattern, YD pattern, and QD pattern, in stroke and the forty-four indices used to determine the pattern^6^. This paper provides the characteristics, laboratory results and symptoms and signs of stroke patients who were treated according to TKM in the SOPI-Stroke project.

Among the symptoms/signs for PI diagnosis, some were common in each of the types of PI (Table 2). Over 80% of subjects with the FH pattern exhibited a reddened complexion, red tongue and a strong pulse. A white “fur” on the tongue and obesity (bi-sup) were major symptoms in the DP pattern, and many patients with the QD pattern appeared powerless and lethargic and had a weak pulse pattern. The YD pattern was associated with paleness, a red zygomatic site and dry mouth. These results were similar to other studies presented by our team and other TCM researchers^13^,^14^.

Many previous studies were performed to evaluate the relationship between Western indicators, such as body composition or blood parameters, and PI patterns^15^,^16^,^17^,^18^,^19^. Tables 3 and 5, respectively, show the distribution of body composition and blood parameters among PI. Among these, the level of obesity indices, including BMI and waist circumference, were significantly higher in the DP and FH patterns than in the QD and YD patterns (Table 3). Total cholesterol, triglycerides and total lipids, which are blood parameters positively related to obesity, were also higher in the DP and FH (Table 5). These results are similar to those previously reported by Min *et al*.^15^, and other studies showed that DP was significantly related to the obese phenotype^16^,^17^,^18^.

Genetic studies showed that many of the gene polymorphisms were related to obesity and obese phenotypes^20^,^21^,^22^,^23^. Similar studies that show the association between genetic polymorphisms and PI were performed in TKM and TCM^17^,^24^,^25^,^26^. Specifically, the polymorphisms in UCP2 and UCP3, which were significantly associated with BMI and serum cholesterol in Korean female subjects, were also associated with DP pattern^24^,^27^,^28^. Some studies showed controversial results. For example, Kim *et al*. reported that subjects with the C allele of −607 G > C in wnt10b had lower BMI levels than subject with the GG type^29^, but Ko *et al*. showed that the −607 G > C polymorphism was related to the YD pattern, not DP pattern^25^. Those results suggested that the PI, a diagnostic system used in TEAM, exhibited similar or different phenotypes compared to the phenotypes discussed in Western medicine.

There are some limitations to our study. To obtain the characteristics of stroke patients treated with TKM, observational studies are very important. This is a potentially interesting observational study because it provided evidence for clinical practice. This paper describes the baseline characteristics of stroke according to PI but there is no follow-up data describing long-term outcomes. Therefore, it is necessary to conduct improved randomized controlled trial studies or cohort studies to obtain reliable results on PI phenotypes and the effect of treatment based on PI compared with Western medicine. Second, the population enrolled in this study focused on the CI type and SVO type according to the TOAST classification. Additionally, the severity of stroke in the enrolled patients was lower than that of stroke patients visiting Western medical hospitals. For this reason, it is difficult to generalize the characteristics of PI. TKM uses four methods of diagnosis, which include diagnosis by observation, hearing and smelling, interrogation, and palpation. Diagnosis depends on the clinician’s experience and knowledge, along with a variety of environmental factors. It is essential to establish an objective diagnostic standard for tongue and pulse measurements, along with other reliable diagnostic tools.

Although this study had some limitations, the significance of our study was to show the characteristics of the patients receiving TKM treatment. In TKM treatment, PI comprises a series of processes that involve not only identifying specific neurological symptoms but also unspecific symptoms and indicators with four examinations, as well as determining treatment goals after integrating all the data for stroke diagnosis. These results should to lead to the standardization of PI for stroke in TKM.

## Methods

### Subjects

**Figure 1:**
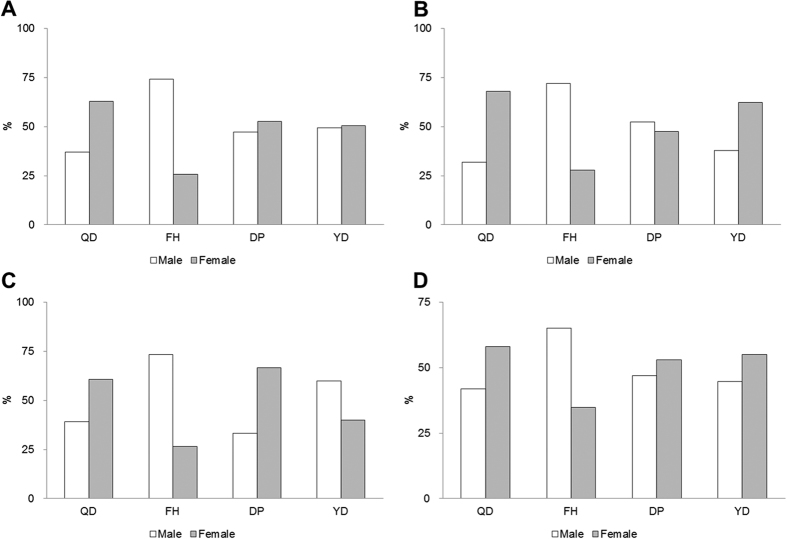
PI distribution among stroke patients by gender according to region. (**A**) Capital (n = 1865): Gachon University Gil Korean Medical Hospital, Kyung Hee Korean Medical Center, Kyung Hee University Hospital at Gangdong, Dong Guk University Hospital, Dong Seo Medical Center; (**B**) Kwang won/Chung cheong(n = 930): Dae Jeon Korean Medical Hospital, Sang Ji Korean Medical Hospital; (**C**) Yeong nam(n = 228): Dae Gu Hanny University Medical Center, Dong Eui Hospital; (**D**) Honam(n = 443): Dong Sin Korean Medical Hospital, Woo Suk University Korean Hospital(Jeonju),Won Kwang Korean Medical Hospital; QD: qi deficiency pattern. FH: fire-heat pattern. YD: yin deficiency pattern. DP: dampness phlegm pattern.

This study was a community-based multicentre trial that was part of the SOPI-Stroke project^5^,^6^. Stroke patients who were admitted to 15 TKM university hospitals participated in this study from 2005 through 2013 (The entire list of hospitals can be found as Supplementary Table S1). Each patient provided written informed consent to undergo procedures that were approved by the respective institutions’ Institutional Review Boards (IRB). This study was conducted in accordance with approved guidelines by the IRB of the KIOM and by each TKM university hospital’s IRB. All patients provided informed consent after a thorough explanation of the details. Figure 1 shows the PI distribution among stroke patients by gender according to region.

### Inclusion/exclusion criteria

We enrolled stroke patients within 30 days of the onset of their symptoms if their diagnosis was confirmed by an imaging diagnosis such as computerized tomography (CT) or magnetic resonance imaging (MRI)^5^,^6^. Patients with traumatic stroke such as subarachnoid, subdural, and epidural haemorrhage were excluded from the study. This study was approved by the IRB of the KIOM and by each TKM university hospital’s IRB.

### Measured variables

Each patient was seen by two experts in the same department within each site. All experts were well trained in standard operation procedures (SOPs). The experts had at least three years of clinical experience with stroke after finishing a regular college education of TKM for six years. The examination parameters were extracted from parts of a case report form (CRF) for the standardization of stroke diagnosis developed by an expert committee organized by the KIOM. The measured variables used included the following key subjects in relation to PI: (a) stroke etiology; (b) distribution of signs/symptoms; (c) physical characteristics and lifestyle parameters; (d) medical history; and (e) laboratory results. Specifically, as suggested by the KIOM, the clinicians were required to measure the stroke PI of each patient according to the FH pattern, DP pattern, QD pattern, or YD pattern.

### Statistics

Data were statistically analysed with SAS software, version 9.1.3 (SAS Institute Inc., Cary, NC, USA). Categorical variables were compared with the chi-square test or Fisher’s exact test, and differences in continuous variables were determined by one-way analysis of variance (ANOVA). Statistical significance was set at P < 0.05.

## Additional Information

**How to cite this article**: Ko, M. M. *et al*. Stroke in Traditional Korean Medicine: A Nine-Year Multicentre Community-Based Study in South Korea. *Sci. Rep.* **6**, 28286; doi: 10.1038/srep28286 (2016).

## Supplementary Material

Supplementary Table s1

## Figures and Tables

**Table 1:** Stroke etiology among pattern identification of study subjects.

Characteristics	Pattern identification					P value
	Total	QD	FH	DP	YD	
Number of patients	3466 (100)	810 (23.37)	1031 (29.75)	1127 (32.52)	498 (14.37)	
Age (yrs), Mean ± SD	67.23 ± 11.49	67.93 ± 12.05	65.94 ± 11.29	66.48 ± 11.11	70.50 ± 11.14	<0.0001
Gender						
Male	1814 (52.34)	297 (36.67)	748 (72.55)	541 (48.00)	228 (45.78)	<0.0001
Female	1652 (47.66)	513 (63.33)	283 (27.45)	586 (52.00)	270 (54.22)	
Stroke subtype						
ICH^1^	302 (8.72)	65 (8.02)	101 (9.80)	86 (7.63)	50 (10.04)	0.0229
SAH^1^	20 (0.58)	11 (1.36)	3 (0.29)	4 (0.35)	2 (0.40)	
CI	3114 (89.92)	723 (89.26)	922 (89.43)	1027 (91.13)	442 (88.76)	
other subtype	27 (0.78)	9 (1.11)	4 (0.39)	10 (0.89)	4 (0.80)	
TOAST classification						
LAA	762 (24.64)	153 (21.16)	252 (27.33)	231 (22.49)	126 (28.51)	0.0167
CE	179 (5.79)	41 (5.67)	59 (6.40)	51 (4.97)	28 (6.33)	
SVO	1990 (64.36)	481 (66.53)	565 (61.28)	684 (66.60)	260 (59.14)	
SOE	46 (1.49)	12 (1.66)	8 (0.87)	17 (1.66)	9 (2.04)	
SUE	111 (3.59)	27 (3.73)	28 (3.04)	39 (3.80)	17 (3.85)	
other subtype	4 (0.13)	3 (0.41)	1 (0.11)	0	0	
Baseline NIHSS score^1^						
<15	2694 (97.57)	563 (97.24)	772 (96.74)	981 (98.30)	378 (97.93)	0.1706
≥15	67 (2.43)	16 (2.76)	26 (3.26)	17 (1.70)	8 (2.07)	
Onset mode of stroke^1^						
On awakening; sleep	644 (22.04)	149 (23.39)	191 (22.90)	211 (20.29)	93 (22.63)	0.4055
Awake	2018 (69.06)	433 (67.97)	577 (69.18)	735 (70.67)	273 (66.42)	
Unknown	260 (8.90)	55 (8.63)	66 (7.91)	94 (9.04)	45 (10.95)	

All results except age are expressed as frequencies (percentages) for categorical variables. QD: qi deficiency pattern. FH: fire-heat pattern. YD: yin deficiency pattern. DP: dampness phlegm pattern. ICH: intracerebral hemorrhage. SAH: subarachnoid hemorrhage. CI: cerebral infarction. TOAST: Trial of ORG 10172 in Acute Stroke Treatment. LAA: large-artery atherosclerosis. CE: cardioembolism. SVO: small-vessel occlusion. SOE: stroke of other etiology. SUE: stroke of undetermined etiology. NIHSS: National Institutes of. Health Stroke Scale.

^1^collected data from June 2005 to April 2011. P values were calculated by ANOVA in continuous variables, chi-squared test in categorical variables.

**Table 2:** Distribution of Korean standard pattern identification for stroke III of study subjects.

	Characteristics	Pattern identification					P value
		Total	QD	FH	DP	YD	
	Number of patients	3466 (100)	810 (23.37)	1031 (29.75)	1127 (32.52)	498 (14.37)	
FH index	Reddened complexion	1074 (64.19)	89 (30.79)	658 (84.35)	206 (53.78)	121 (54.75)	<0.0001
	Headache like flush	184 (16.21)	50 (16.61)	80 (22.03)	26 (8.30)	28 (17.72)	<0.0001
	Heat vexation and aversion to heat	1387 (40.34)	241 (30.08)	562 (55.09)	394 (35.14)	190 (38.30)	<0.0001
	Heat vexation in the chest	464 (29.23)	92 (23.17)	191 (37.45)	106 (25.17)	75 (28.95)	<0.0001
	Vexation and insomnia	496 (29.33)	94 (23.91)	216 (38.70)	113 (24.14)	73 (26.83)	<0.0001
	Thirst	1170 (34.17)	231 (28.66)	433 (42.87)	314 (28.11)	192 (39.10)	<0.0001
	Wheezing in throat with sputum	689 (23.12)	123 (17.47)	219 (24.97)	258 (26.00)	89 (21.92)	<0.0001
	Blood-shot eyes	1570 (45.74)	385 (48.42)	459 (44.82)	510 (45.57)	216 (43.72)	0.3233
	Aphtha or tongue sore	187 (5.83)	34 (4.55)	60 (6.048)	59 (5.86)	34 (7.40)	0.2249
	Vexing heat in the extremities	314 (9.87)	39 (5.27)	137 (14.03)	100 (9.89)	38 (8.35)	<0.0001
	Heat in the palms and soles	318 (9.93)	31 (4.17)	135 (13.69)	90 (8.91)	62 (13.41)	<0.0001
	Turbid urine	769 (26.05)	164 (23.39)	272 (31.81)	221 (23.73)	112 (24.13)	<0.0001
	Fetid mouth odor	718 (21.78)	110 (14.55)	271 (27.07)	258 (24.02)	79 (16.98)	<0.0001
	Yellow fur on tongue	1045 (61.18)	137 (41.76)	458 (71.11)	329 (67.00)	121 (49.38)	<0.0001
	Thick fur on tongue	1238 (57.07)	183 (43.88)	447 (60.32)	506 (68.93)	102 (36.82)	<0.0001
	Red tongue	1164 (69.49)	171 (53.27)	525 (80.52)	219 (56.01)	249 (80.06)	<0.0001
	Strong pulse	1205 (68.97)	116 (47.73)	546 (80.05)	429 (71.26)	114 (51.81)	<0.0001
	Surging pulse	295 (8.72)	24 (3.05)	190 (18.75)	61 (5.53)	20 (4.14)	<0.0001
	Rapid pulse	1030 (65.43)	125 (49.80)	393 (71.32)	333 (65.94)	179 (67.04)	<0.0001
DP index	Sallow complexion	1014 (55.40)	201 (52.48)	147 (33.56)	562 (73.17)	104 (43.15)	<0.0001
	(Physique)heaviness (*bi-sup*)	728 (58.56)	83 (42.13)	211 (56.11)	375 (75.45)	59 (34.10)	<0.0001
	Dark inferior palpebral	515 (14.91)	115 (14.21)	98 (9.57)	240 (21.33)	62 (12.47)	<0.0001
	Dizziness with nausea	644 (18.95)	148 (18.66)	173 (17.12)	230 (20.81)	93 (19.01)	0.1931
	Enlarged tongue	495 (35.50)	113 (34.55)	111 (25.81)	227 (51.70)	44 (22.22)	<0.0001
	White fur on tongue	1754 (78.12)	456 (78.89)	405 (68.64)	698 (88.13)	195 (68.42)	<0.0001
	Slippery pulse	1425 (41.92)	165 (20.96)	365 (35.96)	789 (71.01)	106 (21.81)	<0.0001
QD index	Pale complexion	518 (38.20)	326 (66.39)	49 (13.49)	93 (30.39)	50 (25.51)	<0.0001
	Look powerless and lazy	1713 (49.43)	634 (78.36)	349 (33.85)	461 (40.90)	269 (54.01)	<0.0001
	Feel powerless and lazy	1651 (47.92)	561 (69.68)	366 (35.77)	473 (42.23)	251 (50.50)	<0.0001
	Reluctance to speak	1044 (30.29)	423 (52.61)	216 (21.11)	256 (22.79)	149 (30.04)	<0.0001
	Drowsiness, likes to lie down	954 (35.91)	359 (57.34)	211 (26.74)	249 (28.91)	135 (35.52)	<0.0001
	Reversed cold in the extremities	448 (13.98)	156 (20.82)	88 (9.05)	132 (12.89)	72 (15.68)	<0.0001
	Pale tongue	912 (60.23)	294 (69.01)	154 (38.30)	385 (77.77)	79 (41.36)	<0.0001
	Teeth-marked tongue	487 (34.91)	156 (42.62)	98 (23.38)	186 (44.81)	47 (24.10)	<0.0001
	Weak pulse	931 (61.86)	403 (82.58)	119 (36.06)	246 (57.88)	163 (62.21)	<0.0001
	Fine pulse	671 (19.79)	304 (38.48)	74 (7.31)	110 (9.96)	183 (37.80)	<0.0001
	Slow pulse	552 (44.84)	212 (64.04)	81 (26.38)	203 (49.63)	56 (30.43)	<0.0001
YD index	Pale face and red zygomatic-site	417 (35.36)	53 (20.62)	126 (33.24)	74 (26.81)	164 (61.42)	<0.0001
	Tidal fever	295 (8.63)	53 (6.64)	103 (10.15)	64 (5.75)	75 (15.21)	<0.0001
	(Physique) gauntness (*so-su*)	408 (40.39)	105 (52.5)	131 (38.52)	65 (23.89)	107 (54.04)	<0.0001
	Night sweating	586 (16.94)	120 (14.86)	191 (18.59)	155 (13.76)	120 (24.09)	<0.0001
	Dry mouth	1719 (50.21)	386 (48.06)	545 (53.74)	474 (42.54)	314 (63.82)	<0.0001
	Bare and red tongue like a mirror	156 (14.51)	28 (11.11)	43 (11.81)	11 (4.43)	74 (35.07)	<0.0001
	Dry fur on tongue	676 (37.24)	116 (31.43)	242 (38.59)	165 (33.06)	153 (47.81)	<0.0001

All results are expressed as frequencies (percentages) for categorical variables. The frequency analysis was performed by converting pattern identification index measurements from the 3-point scale in which 3 = very much, 2 = much, or 1 = not much to a 2-point scale (yes or no). P values were calculated by chi-squared test.

**Table 3:** Physical characteristics and lifestyle parameters of study subjects.

Characteristics	Pattern identification					P value
	Total	QD	FH	DP	YD	
Weight (kg), N (Mean ± SD)	3079 (61.66 ± 10.91)	709 (56.92 ± 9.65)	906 (64.79 ± 10.12)	1034 (64.10 ± 10.85)	430 (57.06 ± 10.40)	<0.0001
Height (cm)	2987 (160.69 ± 9.15)	682 (158.15 ± 8.59)	881 (163.77 ± 8.32)	1005 (160.60 ± 8.74)	419 (158.58 ± 10.65)	<0.0001
BMI (kg/m^2^)	2959 (23.82 ± 3.23)	673 (22.67 ± 3.05)	872 (24.16 ± 2.89)	1001 (24.81 ± 3.24)	413 (22.57 ± 3.20)	<0.0001
Waist circumference (cm)	2290 (87.00 ± 9.42)	509 (84.62 ± 8.82)	649 (88.10 ± 9.00)	806 (88.95 ± 9.52)	326 (83.74 ± 9.28)	<0.0001
Hip circumference (cm)	2207 (93.21 ± 8.89)	493 (91.92 ± 8.39)	622 (93.81 ± 8.69)	778 (94.50 ± 8.80)	314 (90.87 ± 9.57)	<0.0001
WHR	2203 (0.935 ± 0.084)	491 (0.923 ± 0.086)	622 (0.942 ± 0.084)	776 (0.941 ± 0.069)	314 (0.928 ± 0.110)	<0.0001
Marital status^1^						
Married/cohabiting	2069 (71.32)	406 (50.12)	660 (64.02)	725 (64.33)	278 (55.82)	<0.0001
Never married	62 (2.14)	12 (1.48)	18 (1.75)	22 (1.95)	10 (2.01)	
Divorced/separated	63 (2.17)	16 (1.98)	20 (2.42)	24 (2.13)	3 (0.60)	
Widowed	707 (24.37)	196 (24.20)	129 (12.51)	263 (23.34)	119 (23.90)	
Stress						
Bereaved of the Spouse	24 (0.70)	8 (0.99)	5 (0.48)	5 (0.44)	6 (1.20)	0.2678
Economic loss	202 (5.68)	37 (4.57)	58 (5.63)	81 (7.19)	26 (5.22)	
Other stress	796 (23.13)	185 (22.84)	241 (23.38)	255 (22.63)	115 (23.09)	
None	2420 (70.31)	575 (70.99)	718 (69.64)	778 (69.03)	349 (70.08)	
Religion						
Yes	1620 (55.84)	401 (63.65)	398 (48.13)	604 (58.41)	217 (52.93)	<0.0001
No	1846 (44.16)	229 (36.35)	429 (51.87)	430 (41.59)	193 (47.07)	
Smoking status						
Current smoker	1010 (29.22)	184 (22.72)	382 (37.05)	316 (28.04)	128 (25.70)	<0.0001
Former smoker	584 (16.90)	106 (13.09)	233 (22.60)	167 (14.82)	78 (15.66)	
Never smoker	1862 (53.88)	518 (63.95)	414 (40.16)	640 (56.79)	290 (58.23)	
Drinking status						
Current drinker	1223 (35.38)	234 (28.89)	460 (44.62)	366 (32.48)	163 (32.73)	<0.0001
Former drinker	381 (11.02)	71 (8.77)	150 (14.55)	110 (9.76)	50 (10.04)	
Never drinker	1853 (53.60)	503 (62.10)	419 (40.64)	647 (57.41)	284 (57.03)	
Exercise						
Yes	1243 (39.17)	286 (35.31)	389 (37.73)	409 (36.29)	159 (31.93)	0.3038
No	1930 (55.68)	443 (54.69)	562 (54.51)	637 (56.52)	288 (57.83)	
Food preference^&^						
Meat (Yes, %)	1448 (44.57)	281 (34.69)	493 (47.82)	487 (43.21)	187 (37.55)	<0.0001
Sea food (Yes, %)	1624 (47.51)	335 (41.36)	519 (50.34)	554 (49.16)	216 (43.37)	<0.0001
Fast food (Yes, %)	1805 (52.89)	440 (54.32)	489 (47.43)	631 (55.99)	245 (49.20)	<0.0001

All results except weight, height, BMI, waist circumference, and hip circumference are expressed as frequencies (percentages) for categorical variables. BMI: body mass index. WHR: waist-hip ratio. ^&^Replied ‘very like ‘or ‘like’.

^1^Collected data from June 2005 to April 2011. P values were calculated by ANOVA in continuous variables, chi-squared test in categorical variables. The sum of N does equal the total number of patients for all variables because some items had missing data.

**Table 4:** Medical history of study subjects.

Characteristics	Pattern identification					P value
	Total	QD	FH	DP	YD	
Number of patients	3466 (100)	810 (23.37)	1031 (29.75)	1127 (32.52)	498 (14.37)	
Past medical history						
TIA (Yes, %)	262 (7.56)	54 (6.67)	75 (7.27)	85 (7.54)	48 (9.64)	0.2918
Facial palsy^1^ (Yes, %)	196 (6.77)	36 (5.74)	68 (8.23)	64 (6.20)	28 (6.91)	0.2220
Hypertension (Yes, %)	2092 (60.67)	475 (58.64)	612 (59.36)	714 (63.35)	291 (58.43)	0.1084
Hyperlipidemia (Yes, %)	466 (13.64)	114 (14.07)	135 (13.09)	166 (14.73)	51 (10.24)	0.1063
Diabetes mellitus (Yes, %)	924 (26.84)	221 (27.28)	270 (26.19)	320 (28.39)	113 (22.69)	0.1148
Ischemic heart disease (Yes, %)	200 (5.85)	54 (6.67)	48 (4.66)	66 (5.86)	32 (6.43)	0.2699
Depression^1^ (Yes, %)	103 (3.56)	35 (5.58)	26 (3.15)	31 (3.00)	11 (2.72)	0.0208
Migraine^1^ (Yes, %)	316 (10.91)	81 (12.86)	98 (11.91)	92 (8.91)	45 (11.08)	0.0569
Stroke^1^ (Yes, %)	590 (20.29)	131 (20.79)	152 (18.31)	225 (21.66)	82 (20.05)	0.3453
Infectious disease^1^ (Yes, %)	304 (10.42)	59 (9.29)	98 (11.78)	81 (7.81)	66 (15.98)	<0.0001
Antithrombotic, anticoagulant therapy (Yes, %)	1125 (33.34)	256 (31.60)	318 (30.84)	378 (33.54)	173 (34.74)	0.3210
(Female) HRT experience^1^ (Yes, %)	46 (3.58)	15 (3.99)	8 (4.15)	14 (2.74)	9 (4.41)	0.6119
(Female) Intake of an oral contraceptive pill^1^ (Yes, %)	5 (0.62)	3 (1.12)	0 (0)	2 (0.63)	0 (0)	0.4676

All results are expressed as frequencies (percentages) for categorical variables. TIA: transient ischemic attack. HRT: hormone replacement therapy.

^1^Collected data from June 2005 to April 2011. P values were calculated by chi-squared test. The sum of N does equal the total number of patients for all variables because some items had missing data.

**Table 5:** Laboratory results of study subjects.

Characteristics	Pattern identification					P value
	Total	QD	FH	DP	YD	
WBC (*10^3^/ml)	3378 (7.52 ± 2.95)	787 (7.2 ± 2.43)	1002 (7.87 ± 3.85)	1111 (7.42 ± 2.40)	478 (7.52 ± 2.62)	<0.0001
RBC (*10^6^/ml)	3313 (4.82 ± 14.48)	771 (4.18 ± 0.53)	987 (5.68 ± 22.91)	1085 (4.75 ± 12.72)	470 (4.26 ± 0.55)	0.1280
Hg (mmHg)	3347 (13.35 ± 1.90)	780 (12.79 ± 1.66)	987 (13.89 ± 2.20)	1103 (13.36 ± 1.68)	477 (13.12 ± 1.76)	<0.0001
Hct (%)	3379 (39.39 ± 8.61)	787 (37.76 ± 4.63)	1003 (41.05 ± 13.81)	1110 (39.36 ± 4.82)	479 (38.67 ± 4.89)	<0.0001
Platelet (*10^3^/ml)	3361 (241.32 ± 82.49)	783 (238.81 ± 84.66)	996 (230.14 ± 82.60)	1103 (251.71 ± 76.95)	479 (244.72 ± 87.85)	<0.0001
Fibrinogen (mg/dL)	1853 (388.70 ± 170.17)	378 (386.07 ± 164.56)	502 (387.10 ± 224.09)	708 (383.70 ± 132.86)	265 (408.89 ± 146.33)	0.2144
Total cholesterol (mg/dL)	3275 (184.72 ± 45.42)	750 (185.23 ± 46.67)	968 (179.94 ± 44.87)	1092 (189.78 ± 45.78)	465 (181.98 ± 42.42)	<0.0001
Triglyceride (mg/dL)	3104 (155.91 ± 110.02)	703 (149.22 ± 124.71)	916 (159.28 ± 103.53)	1060 (163.53 ± 111.04)	425 (140.69 ± 91.83)	0.0008
HDL-cholesterol (mg/dL)	2923 (43.79 ± 17.78)	660 (44.62 ± 13.24)	834 (42.79 ± 14.08)	1020 (43.84 ± 23.54)	409 (44.39 ± 13.73)	0.2039
Total lipid (mg/dL)	834 (537.96 ± 155.26)	146 (525.50 ± 163.70)	203 (524.47 ± 145.35)	399 (560.85 ± 157.84)	86 (484.76 ± 91.83)	<0.0001
Na (mmol/L)	3336 (140.32 ± 5.29)	780 (140.29 ± 6.02)	989 (140.00 ± 5.34)	1096 (140.59 ± 5.26)	471 (140.39 ± 3.73)	0.0913
K (mmol/L)	3336 (4.14 ± 3.43)	780 (4.07 ± 0.51)	988 (4.33 ± 5.43)	1096 (4.11 ± 2.99)	472 (3.96 ± 0.45)	0.1885
Cl (mmol/L)	3270 (104.38 ± 5.68)	742 (104.62 ± 4.27)	979 (104.18 ± 5.94)	1086 (104.46 ± 6.78)	463 (104.24 ± 3.99)	0.4015
GOT (U/ml)	3363 (26.57 ± 22.57)	781 (26.81 ± 36.35)	999 (27.80 ± 17.82)	1107 (25.56 ± 14.99)	476 (25.91 ± 15.48)	0.1274
GPT (U/ml)	3361 (24.54 ± 20.87)	780 (22.91 ± 21.89)	999 (26.75 ± 19.87)	1106 (24.47 ± 21.00)	476 (22.77 ± 20.56)	<0.0001
BUN (mg/dL)	3327 (15.18 ± 6.62)	770 (15.52 ± 6.38)	998 (15.07 ± 5.59)	1091 (14.89 ± 7.64)	468 (15.57 ± 6.47)	0.1117
Cr (mg/dL)	3296 (1.03 ± 6.83)	765 (0.92 ± 0.82)	984 (0.95 ± 0.70)	1082 (1.24 ± 11.88)	465 (0.91 ± 0.69)	0.6868
LDH (IU/L)	2510 (319.32 ± 147.57)	563 (322.30 ± 181.20)	725 (324.21 ± 125.39)	888 (318.99 ± 146.59)	334 (304.54 ± 130.24)	0.2231
CK (IU/L)	2270 (101.13 ± 136.26)	519 (98.13 ± 186.10)	611 (115.72 ± 156.99)	834 (93.28 ± 82.36)	306 (98.51 ± 104.31)	0.0174
FBS (mg/dL)	2610 (118.37 ± 47.71)	560 (114.61 ± 46.55)	730 (117.61 ± 42.39)	934 (120.15 ± 50.90)	386 (120.87 ± 50.63)	0.1081
PP2 (mmol/L)	1446 (170.15 ± 73.21)	271 (166.55 ± 67.59)	406 (163.83 ± 63.41)	537 (172.66 ± 65.28)	232 (179.61 ± 105.18)	0.0421
Homocysteine (umol/L)	1480 (11.32 ± 6.04)	328 (11.22 ± 5.84)	431 (11.50 ± 5.35)	530 (11.24 ± 6.61)	191 (11.28 ± 6.25)	0.0281
Vitamine B12 (pg/mL)	938 (663.06 ± 370.32)	162 (684.92 ± 403.21)	270 (611.33 ± 333.16)	403 (673.40 ± 374.10)	103 (723.79 ± 383.28)	<0.0001
Folic acid (ng/mL)	946 (9.14 ± 30.54)	166 (11.92 ± 55.42)	275 (9.13 ± 35.97)	400 (8.24 ± 6.14)	105 (8.18 ± 5.39)	0.6098

All results are expressed as N (Mean ± SD). ^1^Collected data from June 2005 to April 2011. WBC: white blood cell. RBC: red blood cell. Hg: hemoglobin. Hct: hematocrit. GOT: glutamate oxaloacetate transaminase. GPT: glutamate pyruvate transaminase. BUN: blood urea nitrogen. Cr: creatinine. LDH: lactate dehydrogenase. CK: creatine kinase. FBS: fasting blood suger. PP2: 2 hours postprandial blood. P values were calculated by ANOVA. The sum of N does equal the total number of patients for all variables because some items had missing data.
