# Supplementary material for: Stroke in Traditional Korean Medicine: A Nine-Year Multicentre Community-Based Study in South Korea
Source: Sci Rep. 2016 Jun 22;6:28286. doi: 10.1038/srep28286 (PMC4916442; doi:10.1038/srep28286)
Supplement: Supplementary Table s1 [file srep28286-s1.pdf]

Stroke in Traditional Korean Medicine: A Nine-Year Multicentre Community-Based Study in South Korea

By *Mi Mi Ko, Ju Ah Lee, Min Ho Cha , Byoung-Kab Kang, and Myeong Soo Lee*

Supplementary Table S1. The number of stroke patients according to hospitals that participated in this study from 2005 to 2013

Hospital(region)	year								
	~2006	2007	2008	2009	2010	2011	2012	2013	total
Kyung Won Korean Medical Hospital(Seoul)[$]		18	22	1					41
Kyung Won Korean Medical Hospital(Incheon)[$]		56	62	47	35				200
Kyung Hee Korean Medical Center(Seoul)		235	410	314	190	101	62	64	1376
Kyung Hee University Hospital at Gangdong(Seoul)		1	106	173	140	31	22	20	493
Dae Gu Hanny University Medical Center(Daegu)			8	48	30				86
Dae Jeon Korean Medical Hospital (daeheung-dong, Daejeon)l	32	236	191	166	163	44	18	33	883
Dae Jeon Korean Medical Hospital (dunsan-dong, Daejeon)					25	33	33	22	113
Dong Guk University Hospital(Kunggido)			54	232	45				331
Dong Guk University Korean Hospital(Kunggido)		139	109	48					296
Dong Seo Medical Center(Seoul)	10	87	39	8					144
Dong Sin Korean Medical Hospital(Gwangju)		46	39	42	43				170
Dong Sin Korean Medical Hospital(Suncheon)		43	8						51
Dong Eui Hospital(Pusan)		43				66	42	49	200
Sang Ji Korean Medical Hospital(Wonju)		58	35	26	25				144
Woo Suk University Korean Hospital(Jeonju)		27	16	12					55
Won Kwang Korean Medical Hospital (Iksan)	2	59	21	24					106
Won Kwang Korean Medical Hospital (Jeonju)		77	62	60	33				232

total	44	1125	1182	1201	729	275	177	188	4921

[$]Gil Korean Medical Hospital, Gachon University
